# Extended Half-Life Coagulation Factors: A New Era in the Management of Hemophilia Patients

**DOI:** 10.4274/tjh.galenos.2019.2018.0393

**Published:** 2019-08-02

**Authors:** Muhlis Cem Ar, Can Balkan, Kaan Kavaklı

**Affiliations:** 1 İstanbul University-Cerrahpaşa, Cerrahpaşa Faculty of Medicine Department of Internal Medicine, Division of Hematology, İstanbul, Turkey; 2 Ege University Faculty of Medicine, Department of Pediatrics, Division of Hemato-Oncology, İzmir, Turkey

**Keywords:** Hemophilia, Factor replacement therapy, Extended half-life products, Laboratory assays, Pharmacokinetics, Quality of life

## Abstract

Despite effective factor replacement and various treatment schedules, there remain several challenges and unmet needs in the prophylactic treatment of hemophilia limiting its adoption and thereby posing an increased risk of spontaneous bleeding. In this regard, extended half-life (EHL) recombinant factor VIII (rFVIII) and factor IX (rFIX) products promise optimal prophylaxis by decreasing the dose frequency, increasing the compliance, and improving the quality of life without compromising safety and efficacy. EHL products might lead to higher trough levels without increasing infusion frequency, or could facilitate the ability to maintain trough levels while reducing infusion frequency. This paper aims to provide a comprehensive review of the rationale for developing EHL coagulation factors and their utility in the management of hemophilia, with special emphasis on optimal techniques for half-life extension and criteria for defining EHL coagulation factors, as well as indications, efficacy, and safety issues of the currently available EHL-rFVIII and EHL-rFIX products. Potential impacts of these factors on quality of life, health economics, and immune tolerance treatment will also be discussed alongside the challenges in pharmacokinetic-driven prophylaxis and difficulties in monitoring the EHL products with laboratory assays.

## Abstract (tr)

Etkin faktör yerine koyma tedavisi ve değişik tedavi programlarına rağmen günümüzde hemofilinin profilaktik tedavisinde hala çözülmemiş sorunlar ve karşılanmamış gereksinimler vardır. Bu nedenle kanama riski ve kanamaya bağlı komplikasyonlar önemini korumaktadır. Bu bağlamda profilakside kullanılacak uzatılmış yarı ömürlü rekombinant faktör VIII ve faktör IX ürünleri tedavinin güvenlilik ve etkililiğinden ödün vermeksizin doz sıklığının azaltılması, hasta uyumunun artması ve yaşam kalitesinin düzelmesini sağlayarak optimal tedavi koşullarının oluşmasına yardımcı olabilir. Uzatılmış yarı ömürlü faktörler infüzyon sıklığını artırmadan daha yüksek çukur değerler ulaşılması veya mevcut çukur değerin daha seyrek infüzyonla idamesi konusunda önemli bir açılım sağlayabilir. Bu derlemede uzatılmış yarı ömürlü faktör konsantrelerinin geliştirilmesine neden gerek duyulduğu, hemofili tedavisindeki olası yerleri, faktör yarı ömrünü uzatmak için kullanılan teknikler ve mevcut uzatılmış yarı ömürlü faktör konsantrelerinin etkililik, güvenlilik ve endikasyonları ile ilişkili kapsamlı bilgi sunulacaktır. Ayrıca bu ürünlerin yaşam kalitesi ve sağlık ekonomisi üzerine etkileri, immün tolerans tedavisindeki yerleri, farmakokinetik temelli profilaside kullanımları ile laboratuvar izleminde karşılaşılan güçlükler tartışılacaktır.

## Introduction

Hemophilia A and B are X-linked monogenic inborn coagulation defects that lead to deficiencies of factor VIII (FVIII) and factor IX (FIX) in approximately 1 of 5000 and 1 of 30,000 male live births, respectively [1,2,3].

The disease phenotype is characterized by recurrent spontaneous or traumatic bleeding episodes predominantly involving the weight-bearing joints, skeletal muscle, and soft tissues. Intracranial and retroperitoneal hematomas are rare but life-threatening complications of severe hemophilia [1,3]. The bleeding phenotype has been defined as “severe”, “moderate”, and “mild” based on the level of the residual endogenous factor being <1 IU/dL, 1-5 IU/dL, and 5-40 IU/dL, respectively [4,5].

Replacement of the missing factor constitutes the mainstay of hemophilia treatment. Factor replacement is given either on demand to treat acute bleeding or prophylactically to prevent bleeding [2,6]. In severe hemophilia, recurrent bleeding, typically in the form of joint bleeds and skeletal muscle hematomas, results in progressive hemophilic arthropathy and muscle contractures, which eventually lead to irreversible joint damage, significant disability, and decreased quality of life unless treated with FVIII and FIX [4,5,6,7,8].

Regular prophylactic factor replacement to maintain circulating factor levels of >1 IU/dL (1%) has been recommended as the optimal therapy for people with severe hemophilia, based on evidence showing that prophylaxis is associated with substantial reduction in bleeding episodes and related complications and consequently with an improvement in the quality of life and life expectancy [9,10,11].

In the prophylactic setting, people with severe hemophilia A usually require intravenous injections three times a week, while those with severe hemophilia B are usually treated twice weekly, owing to the longer half-life of FIX compared to FVIII (18-20 h vs. 8-12 h) [2,12].

Due to the relatively short half-life of conventional factor concentrates, frequent intravenous administrations are required to maintain plasma factor levels above the target threshold level to avoid bleeding, and this necessitates frequent injections [13,14]. The requirement for frequent dosing not only creates venous access problems but also poses an obstacle to patient adherence and proper use and adoption of prophylaxis [15,16,17,18]. This, in turn, may lead to treatment failure, resulting in increased disability [19,20,21,22,23]. Hence, there is an unmet need for factor concentrates with longer half-lives that would allow for a more successful prophylaxis at less frequent dosing [23,24,25] and would consequently result in reduced prophylactic treatment burden for patients and caregivers.

Much effort has been devoted to the optimization of the pharmacokinetics (PKs) of recombinant factors by molecular modifications to achieve extended half-life (EHL) FVIII and FIX products [3,23,26,27]. The new-generation EHL factor concentrates are expected to facilitate the implementation of optimal prophylaxis, allowing longer treatment intervals without loss of efficacy. Treatment with EHL factors would reduce the burden of frequent intravenous interventions, enable higher adherence to treatment, and improve quality of life [4,28,29,30,31].

During the past decade numerous techniques have been invented for the development of EHL-rFVIII and -rFIX molecules, all of which principally exert their effect by decreasing the clearance of the factors. A combination of reduced proteolysis in peripheral blood, decreased renal and hepatic elimination, and decreased receptor-mediated endocytosis usually results in prolongation of the half-life of the factor [32]. Several novel EHL-rFVIII and -rFIX products have entered the market or are about to launch following the completion of their phase 3 studies [33,34,35,36,37,38,39,40,41].

This paper aims to provide a comprehensive review of the rationale for developing EHL coagulation factors and their utility in the management of hemophilia, with special emphasis on optimal techniques for half-life extension and criteria for defining EHL coagulation factors, as well as indications, efficacy, and safety issues of the currently available EHL-rFVIII and EHL-rFIX products. Potential impacts of these factors on quality of life, health economics, and immune tolerance treatment will be also be discussed alongside the challenges in PK-driven prophylaxis and difficulties in monitoring EHL products with laboratory assays.

## Evolution of Factor Replacement Therapy

Management of hemophilia mainly depends on replacing the missing coagulation factor to stop (episodic or on-demand therapy) or to prevent (prophylaxis) bleeding episodes [4,5]. The concept of prophylaxis is based on early experiences with patients having mild to moderate hemophilia (factor levels >1%) who bled less frequently and rarely developed arthropathy [7]. Prophylaxis has been considered as the gold standard for the management of hemophilia as it prevents bleeding and delays development of joint damage by providing sufficient levels of the missing factor [17,42]. Several prospective studies have definitively shown the superiority of prophylaxis compared to on-demand treatment in reducing the frequency of joint bleeds and hemophilic arthropathy, and in improving the quality of life [17,43,44,45,46]. Since 1994 prophylaxis with coagulation factors has been regarded as the standard of care for the management of hemophilia. Early implementation of prophylaxis might prevent the development of arthropathy in children and might slow down the progression of established arthropathy in adults [5,47,48].

## EHL Coagulation Factors: An Unmet Need

FVIII and FIX are large, complex proteins with relatively short half-lives, necessitating frequent dosing to maintain therapeutic levels. EHL coagulation factors are designed to have prolonged half-lives through some structural modifications, such as chemical alterations or fusion of the factor protein to another molecule with a longer half-life. In theory, an extended half-life product is expected to result in better adherence to treatment and improved prophylactic outcomes by allowing for less frequent injections [49].

The optimal method for half-life extension should not cause any change in the biological activity and safety of the coagulation factor [49]. The definition of a clinically relevant extension of half-life is usually based on some practical criteria such as the dosing schedule and the intended clinical application (e.g., on-demand vs prophylaxis) [49]. The lower clearance rate of EHL factors provides potential for reducing treatment burden with less frequent injections and equal or improved efficacy without increasing the overall factor consumption [14]. This allows greater flexibility for individualizing prophylaxis according to the needs of the patient, leading to better adherence and consequently improved standard of care in hemophilia [14,50].

## Technologies Used for Extending the Half-Life of Recombinant Clotting Factors

The strategies to prolong the half-life of recombinant coagulation factors include *i*) covalent attachment of the coagulation factor to polyethylene glycol (PEG; PEGylation) to reduce interaction with clearance receptors; *ii*) integrating the coagulation factor with the fragmented crystallizable (Fc) portion of the immunoglobulin G1 (IgG1) molecule to divert the molecule away from lysosomal degradation to delay its clearance or *iii*) combining the coagulation factor and recombinant albumin, to rescue endocytosed proteins from the intracellular degradation pathway; and *iv*) single-chain technology for augmenting the stability of the molecule [4,19,51,52].

### *PEGylation*

The pharmacokinetic and pharmacodynamic properties of the coagulation factors can be changed through PEGylation, which involves the covalent binding of PEG to FVIII or FIX [19]. The circulating half-life of the PEGylated factor concentrates is increased through PEGylation, which decreases the binding potential of the PEGylated proteins to their clearance receptors and consequently reduces their degradation [19,52]. Overall, PEGylation of therapeutic molecules has generally been associated with a low risk of immunogenicity [19]. The preclinical study results of the extended half-life product BAY 94-9027 suggested that this compound was significantly less immunogenic in hemophilia A mice, normal rats, and normal rabbits when compared to the un-PEGylated rFVIII. However, human data are lacking [53].

PEGylated factors have been reported to have a half-life prolongation of about 1.5-fold when compared to the standard half-life (SHL) factors. Excellent safety and efficacy data in previously treated adults and children with severe hemophilia A have been reported with EHL factors. No inhibitory antibodies have been identified to FVIII, the PEGylated product, or to PEG [4,29,33,54,55,56]. The development of anti‐PEG antibodies in patients treated with other PEGylated protein products has been reported, which led the FDA to recommend the screening of anti-PEG antibodies in all subjects receiving experimental PEGylated therapeutics, as well as the evaluation of the potential roles of these antibodies on efficacy and safety [57,58,59].

### *Fusion Protein Technology (Fc Fusion and Albumin Fusion)*

Fusion protein technology involves genetic fusion with a protein that has a particularly long half-life, such as immunoglobulins (Fc fusion) or albumin [28,60,61]. Albumin and IgG are naturally occurring proteins with long half-lives (exceeding 20 days) and account for about 80% of the proteins in plasma, making them useful tools for fusion protein technologies [52]. The prolonged half-lives of albumin and IgG are a result of neonatal Fc receptor (FcRn)-mediated recycling, a naturally occurring recycling pathway that prolongs the half-lives of various proteins by diverting them away from lysosomal degradation, which leads to delayed clearance and extended functional plasma half-lives [19,49,52].

Albumin fusion technology has recently been utilized for the prolongation of the half-life of rFIX [28]. The mean half-life extension of rFVIII-Fc is about 1.5-fold that of SHL-FVIII [4,29]. The modest increase in the half-life of FVIII when attached to albumin as compared with the fivefold half-life extension seen with FIX when fused with Fc has been considered to be mainly due to the interaction of FVIII with von Willebrand factor (VWF) as the main regulator of FVIII clearance [54,62].

The long-term efficacy and safety of rFVIII-Fc in the management of bleeding and prophylaxis of previously treated patients (PTPs) with severe hemophilia A have been confirmed by several studies, including pivotal phase III trials (A-LONG) done in adults and adolescents aged >12 years, the Kids A-LONG trial performed in children aged ≤12 years, and ASPIRE, a recently completed extension study [4,34,63,64].

### *Single-Chain Technology*

Human FVIII is a heterodimeric structure consisting of a heavy chain (A1-A2-B domains) and a light chain (A3-C1-C2 domains) attached to each other by noncovalent bonds, which makes it relatively unstable and easily dissociable to inactivated FVIII chains [4,23,49]. A novel recombinant single-chain FVIII (rVIII-SingleChain) has been engineered, in which the heavy and light chains are covalently bound through a truncated B domain [4,65,66]. This single-chain design has been reported to yield a more stable and homogeneous product, with increased binding affinity for VWF and improved PKs relative to the full-length rFVIII, potentially prolonging the half-life of FVIII [4,23,29]. Although rVIII-SingleChain was well tolerated in clinical studies and did not lead to the development of inhibitory antibodies, the extension of the half-life using this technology was modest, being 1.1- to 1.4-fold of the original FVIII half-life [65,66,67].

## Currently Available EHL-rFIX and EHL-rFVIII Products

Three EHL-rFIX products have completed phase 3 clinical studies and are licensed for adolescent and adult patients [26,41], including rFIXFc (Alprolix, Sobi, Stockholm, Sweden; Bioverativ, a Sanofi company, Waltham, MA, USA) [37], nonacog beta pegol (N9-GP, Novo Nordisk A/S, Bagsværd, Denmark) [39], and rFIX-FP (Idelvion, CSL Behring, King of Prussia, PA, USA) [38] (Table 1).

There are four EHL-rFVIII products that have completed phase 3 clinical studies. rFVIIIFc (Elocta, Sobi, Stockholm, Sweden; Eloctate, Bioverativ, Waltham, MA, USA) [34] and octocog alfa pegol (Adynovate, BAX 855, Baxalta, Vienna, Austria) [33] are licensed in some countries. Turoctocog alfa pegol (N8-GP, Novo Nordisk A/S, Bagsværd, Denmark) [35] has completed a phase 3 study and phase 2/3 data were published for BAY 94-9027 (Jivi, Bayer Healthcare AG, Leverkusen, Germany) [56]. Finally, a B-domain-truncated single-chain rFVIII concentrate, ScrFVIII (Afstyla), has recently been licensed by CSL Behring in some countries [40]. However, this product displays a modest extension in half-life and is not regarded as an extended half-life concentrate (Table 1).

Considering the mechanisms of half-life extension in rFIX concentrates, rFIX-Fc (Alprolix) fuses the Fc immunoglobulin region with FIX, while rFIX-FP (Idelvion) combines FIX with albumin and N9-GP (Rebinyn/Refixia) is a PEGylated version of FIX. As with EHL-rFIX concentrates, EHL-rFVIII concentrates are also based on Fc fusion (rFVIIIFc, Elocta/Eloctate) or PEGylation (N8-GP; BAX 855 and BAY 94-9027). BAY 94-9027 and N8-GP are B-domain-deleted (BDD) rFVIII, whereas BAX 855 is a full-length rFVIII [6,41,56,68] (Table 1).

The main characteristics of EHL concentrates are provided in Table 2. For all three EHL-rFIX, an unmodified rFIX protein was used and an increase in the extension of rFIX half-life (3.8-fold, ranging from 2.4- to 4.8-fold) and extension in the dosing frequency for prophylaxis (ranging from 7 to 14 days) were evident when compared to the SHL-FIX [26]. Management of bleeding episodes as well as prophylactic replacement therapy with all three EHL-rFIX products have been reported to be successful, providing evidence for high overall hemostatic activity. Bleeding episodes could effectively be treated with 1 or 2 injections. A consistent decrease in clearance and increased area under the curve (AUC) as well as an increased incremental recovery were noted for all three EHL-rFIX for the same dose of 50 IU/kg of EHL-rFIX in comparison to SHL-rFIX, leading to substantial and meaningful prolongations of half-life and justifying a once weekly dosing regimen. Overall, the safety profiles of all three EHL-rFIX products were satisfactory in the adolescent and adult setting, with no signs of inhibitor development or drug-related serious adverse events (Table 2).

The half-life extension of EHL-rFVIII products is in the range of 1.4- to 1.6-fold and the annualized bleeding rates (ABRs) were below 4, ranging from 1.3 to 3.6. Bleeds were treated successfully with EHL-rFVIII, resolving with one or two injections in more than 96% of episodes. Hemostatic efficacy was rated as good or excellent in more than 90% of the bleeding episodes. No inhibitor development has been reported in clinical trials with the EHL-FVIII products (Table 2).

Data on the use of EHL products in the pediatric age group [64,69,70,71] revealed low median ABRs ranging from 1 to 3 bleeds, with no major difference between the products and no inhibitor development [26]. The modest prolongation achieved in the half-life of the FVIII products achieved through extension techniques could only reduce the treatment frequency to twice weekly.

The safety and efficacy as well as the PK profile of rVIII-SingleChain have been studied within the framework of a clinical trial program called AFFINITY, consisting of a series of phase I/III studies [29,66,72]. Preliminary data from this program showed excellent/good hemostatic efficacy in both prophylactic and episodic treatment with a good safety profile. No inhibitor development has been reported to date with rVIII-SingleChain [72]. PK analysis showed a favorable PK profile for rVIII-SingleChain compared to full-length rFVIII, though the half-life was relatively shorter and the clearance relatively higher in the pediatric group [73].

## Criteria for Classifying a Replacement Factor as an EHL Product

The advent of EHL recombinant factors has been an important evolution in concentrate manufacturing, providing a new treatment tool for individualized hemophilia care [41,74,75]. However, as is the case with every new treatment option that enters the market, the introduction of EHL coagulation factors has also raised concerns regarding their optimal utility to provide the best possible outcome for each patient [52].

Ideally, EHL recombinant factors should allow reduced dosing frequency with retention of hemostatic efficacy compared to SHL recombinant factors for the majority of patients [76]. However, the current literature does not provide clarity regarding the definition of EHL and SHL products [76].

Given the tight non-covalent association of FVIII with VWF in the circulation, which imposes a biological limit on the extension of the half-life of FVIII beyond that of VWF, the EHL-rFVIII products have not had an equally substantial improvement in half-life as observed with EHL-rFIX products [76,77]. EHL-rFIX products demonstrated a 3- to 5-fold increase in half-life compared to standard FIX concentrates, providing a clear threshold for differentiating the EHL products from the standard ones [76,78].

However, this is not always the case for FVIII products. In a modeling study designed to identify the minimum half-life extension ratio required for a reduction in dosing frequency while maintaining the proportion of patients with plasma rFVIII levels >1 IU/dL with no increase in the total weekly dose, the authors found that a meaningful reduction in the infusion burden of an EHL-rFVIII product (relative to a standard rFVIII) is only possible when the half-life extension ratio is ≥1.3 [79]. In addition, it has been suggested that both the AUC ratio and the half-life ratio should be used to provide sufficient PK evidence for a solid definition of EHL [76]. Accordingly, “EHL-rFVIII” designation requires the fulfillment of the following 3 criteria: *i*) the product should be designed and engineered with relevant technology used to extend the circulating biological half-life; *ii*) the difference from the SHL-rFVIII comparator should be demonstrated for the majority of patients according to the proposed “bio-difference” criterion based on the lower limit of the 90% CI for the AUC ratio being above the FDA/EMA cut-off for bioequivalence (1.25 or 125%); and *iii*) a half-life ratio of 1.3 or higher, based on modeling, should be achieved [76,79].

BAX 855 and rFVIIIFc have been reported to clearly comply with all 3 of these criteria, while rFVIII-SingleChain failed to fulfill the criteria since it cannot be fully differentiated from the standard rFVIII (Advate^®^), based on the 90% CIs for the AUC ratio extending below 1.25 and a half-life extension ratio of 1.09 when compared to Advate^®^. This suggests that rFVIII-SingleChain may behave like standard rFVIII in some patients despite its modified PK characteristics [76]. Although there are some limitations imposed by the different study designs and reporting, current evidence suggests that both BAY 94-9027 and N8-GP fulfill the criteria for EHL-rFVIII, signifying that they should be classified as EHL-rFVIII products [76].

Definition and classification are always of help for a better understanding of the potential benefits and limitations of recombinant factor products. However, one should never forget that these cannot substitute for careful clinical monitoring of patients, including measurement of rFVIII levels and individual PK profiles.

## Indications and Utility of EHL: Switching from SHL Factors to EHL Factors

The published phase I-III studies on prophylaxis with EHL-rFVIII and -rFIX products revealed improved PK profiles with prolonged half-lives ranging from 1.2- to 1.5-fold for FVIII and 3- to 5-fold for FIX [19]. EHL products were shown to be well tolerated with no inhibitor development in the PTP population. They were efficacious in the treatment and prevention of bleeding episodes with the potential to reduce the infusion load and to achieve higher trough levels [19,74,76].

The market availability of effective EHL products with the potential of reducing infusion frequency will inevitably induce a transition from SHL to EHL factor concentrates, in both episodic treatment and prophylactic settings. Extension of half-life might lead to higher trough levels without increasing infusion frequency, or could facilitate the ability to maintain trough levels while reducing infusion frequency. Either of these strategies could be implemented to improve outcomes, depending on the characteristics of the patient [74,75]. In a study using dosing simulations to investigate potential clinical outcomes via different prophylactic regimens with rFVIIIFc and rFVIII, the authors suggested that patients with different needs might benefit in different ways from transitioning from rFVIII to rFVIIIFc [14]. A high correlation of PK data between rFVIIIFc and rFVIII was also noted with a one-third lower average clearance for rFVIIIFc, which could be useful for adjusting doses in the case of a transition between the two products [14].

Accordingly, “standardized” (dose and interval fixed to once weekly for FIX and twice weekly for FVIII), “PK-driven” (dosed to a target trough, fixed interval), “phenotype-driven” (variable dose and interval according to bleeding pattern and activity), and “convenience-driven” (higher dose, longer interval) strategies have been used for the prophylaxis regimens in pivotal clinical trial programs [19].

There might be a concern regarding inhibitor development when switching between different FVIII concentrates as product type is one of treatment-related factors for inhibitor development [77]. Recent real-world data on EHL factor concentrates are in support of the data obtained from previous clinical studies with these products in PTPs, stating that no inhibitor formation was observed in patients who switched from conventional factor VIII or IX replacement to treatment with EHL-rFVIII or -rFIX [78,79,80]. In non-adherent patients, switching to a standardized prophylaxis regimen with EHL factors (once or twice weekly) has been associated with a successful treatment outcome leading to trough levels sufficient to suppress target joint bleeding [19]. Patients who were bleeding under conventional rFVIII treatment have been shown to benefit both from improved bleeding control and reduced injection frequency when switched to rFVIIIFc prophylaxis with similar prophylactic factor consumption [14,84]. Thus, the same total weekly prophylactic dose might be given initially, in divided daily doses, twice weekly instead of 3 times a week when switching from SHL-FVIII to rFVIIIFc. Thereafter, the dose and dosing interval can be adjusted depending on the patient’s clinical needs [14].

Accordingly, data from the ALONG trial showed that 30% of hemophilia A patients in the individualized prophylaxis arm achieved 5-day dosing intervals in the last 3 months of the study [34]. In the ASPIRE trial, the phase 3b extension study, interim data revealed that further prolongation of dosing intervals to 7-day intervals was possible in 2 of the 33 patients who were on twice weekly dosing and 10 of 37 who were on every-5-day dosing in the ALONG study [59]. Overall, median ABRs were lower with rFVIIIFc prophylaxis (individualized prophylaxis: 0.66, weekly prophylaxis: 2.03; modified prophylaxis: 1.97) as compared with on-demand treatment (18.36) [63].

A 30% lower total weekly dose of rFVIIIFc has been shown to be likely to give the same FVIII exposure considering that rFVIIIFc has a 30% lower clearance when compared to rFVIII [14]. Based on this, one can conclude that patients on prophylactic treatment who are well controlled with a conventional FVIII product might maintain the same level of bleeding control while benefiting from reduced injection frequency and decreased prophylactic factor consumption when switched to EHL-rFVIII. However, this has to be further investigated in clinical trials.

While the primary focus has been the potential use of EHL factor concentrates in patients with severe hemophilia, the utility of EHL products for the management of bleeding in patients with mild and moderate deficiencies has also been considered [52].

Further support with real-life data on the potential advantages of EHL factor concentrates may enable identifying the needs and characteristics of individual patients and the difference in the behavior of SHL-FVIII products as compared to EHL products. This may help guide clinicians when switching hemophilia A patients from conventional FVIII to EHL products.

Nonetheless, it should be noted that commenting on the comparative efficacy of new long-acting therapies is not possible due to the lack of head-to-head studies. Furthermore, it is difficult to compare different EHL products with the current clinical data since the published studies greatly vary with regard to study populations, study designs, and protocols and they evaluate outcomes using different end-points such as ABR, inhibitor development, number of breakthrough bleeds, or dosing intervals.

## Current Evidence on the Impact of Quality of Life and Health Economics

Prophylaxis is considered as the gold standard for countries in which it is economically affordable [41,85,86], and it is associated with a better health-related quality of life (HRQoL) as compared to episodic treatment [87]. Furthermore, it leads to a decrease in bleeding-related hospitalization, shortens length of hospital stay, and thus reduces resource utilization [85,88].

EHL factors offer better protection from bleeding while reducing the frequency of injections and allowing trough activity levels to remain above key thresholds for longer periods relative to conventional factor products [19]. Thus, longer half-lives and reduced clearance of EHL factors are suggested to result in reduced factor consumption while maintaining higher trough levels and leading to improved protection from bleeding. This, in turn, causes a considerable reduction in hemophilia-related complications and their associated cost burden [19].

In two phase III studies done with rFVIIIFc and rFIXFc, HRQoL was assessed in adults with severe hemophilia A and B, respectively, who received prophylactic or episodic factor replacement regimens [34,37]. The post hoc analyses of these studies revealed that prophylaxis with rFVIIIFc or rFIXFc was associated with significant improvements in HRQoL (particularly in ‘Physical Health’ and ‘Sports and Leisure’ domains) over time. This has also been noted in patients who had been receiving prophylaxis with SHL-rFVIII/FIX and were switched to EHL-rFVIIIFc and -rFIXFc. Thus, EHL factor concentrates seem to further improve the HRQoL of hemophilia patients [87].

In an analysis of the potential financial consequences of introducing rFVIIIFc to a private payer system in the United States, rFVIIIFc was anticipated to have a budget impact of 1.4% across 2 years for a private payer population of 1,000,000 through reducing the ABR by approximately 3.1 bleeds per individual patient with hemophilia A [85]. The total population budget was predicted to decrease for episodic treatment with the introduction of rFVIIIFc based on the lower factor consumption data observed in the ASPIRE trial [63,85].

For prophylaxis, the cost per bleed avoided after the introduction of rFVIIIFc was estimated to be 1974$ in year 1 and 1808$ in year 2, while the small decrease in cost per bleed avoided over time was considered to be associated with the likelihood of patients uncontrolled on a fixed prophylaxis regimen to switch to an individualized regimen in year 2, resulting in a more efficient use of factor therapy [85].

In the scenario without rFVIIIFc, the annual cost for the management of the estimated annual 388 bleeding episodes was 2,044,868$, which equates to each bleeding episode costing approximately 5270$. The cost per bleed avoided with rFVIIIFc on the market was approximately 1891$, indicating that prophylaxis with rFVIIIFc provides good value for money in the prevention of joint bleeds and related comorbidities [85].

The introduction of rFVIIIFc to a private payer system is anticipated to have a minimal budget impact while reducing the ABR, alongside a potential for reduced dosing schedule required for rFVIIIFc and reduction in total factor consumption, facilitating adherence to prophylaxis regimens with a likely positive impact on patient quality of life and economic burden [85].

## Role of EHL Factors in Immune Tolerance Treatment

Occurring in up to 30% of patients with severe hemophilia A, the development of alloantibodies (inhibitors) directed against FVIII is the most serious complication of replacement therapy [2], leading to treatment failure, preventing patients from receiving long-term prophylaxis, and exposing them to an increased risk of mortality, morbidity, and disability [2,6,89].

The current management of patients with inhibitor development involves treating acute bleeding with agents that bypass the need for FVIII or FIX, i.e. activated prothrombin complex concentrate or activated recombinant factor FVII, and using immune tolerance induction (ITI) in order to eradicate the inhibitory antibodies [89]. Several protocols of ITI have been released with overall success rates of about 70% [89,90].

While several meta-analyses failed to demonstrate a significant difference of inhibitor development in patients treated with recombinant FVIII compared to plasma-derived products, a randomized prospective study revealed that the use of recombinant products in previously untreated patients (PUPs) was associated with a 1.8 times greater risk of inhibitor development compared to plasma-derived products [91]. There is no published study to date presenting definitive results with novel EHL concentrates in PUPs, while all the studies in PTPs showed an excellent safety profile with substantially no inhibitor development after switching to novel products [6]. Nonetheless, at the immunogenicity level, so far, available data from PTP clinical trials suggest that EHL factors are safe, with no increased risk for inhibitor development [34,64,89].

A potential role for EHL factors for the induction of immune tolerance has also been suggested [19,52]. A series of case reports regarding the use of EHL-rFVIII in ITI described the successful treatment of children with severe hemophilia A and high-titer inhibitor using different doses of rFVIIIFc in ITI ranging from 50 to 200 IU/kg per dose [92,93]. Hence, with no reports of any EHL factors causing inhibitor formation in the initial clinical trials and lack of randomized trials in PUPs and in ITI, the rationale of safely using rFVIIIFc should depend on case reports and strong laboratory data [52,89]. The available evidence encourages the consideration of the use of rFVIIIFc to eradicate inhibitors, particularly in refractory patients and those with a high-risk profile (i.e. those with a family history of failure of ITI with standard factors or history of a high-peak inhibitor) [52,89].

## Challenges in Pharmacokinetic-Guided Prophylaxis with EHL Products

Prophylactic dosing in severe hemophilia is generally tailored according to the individual needs of the patients. Tailoring of treatment has been guided by either clinical bleeding phenotype or individual PKs of a particular factor concentrate in a patient [19].

PK-tailored prophylaxis was shown to have superior hemostatic efficacy compared to on-demand treatment, along with decreased factor consumption [17]. PK-tailored dosing, explored in several of the phase 3 clinical trials with EHL factors, was associated with good efficacy in bleeding control in these prospective studies [19]. Hence, all licensed EHL products recommend tailoring the dose to the individual patient’s PK response, since standardized dosing may result in patients being undertreated if factor clearance is higher than expected [41].

Individualized PK-based dosing is considered as an alternative option for maintaining a predetermined factor trough level [94,95]. However, given the burden and cost of frequent blood sampling required for personalized PK assessment and the likelihood of a lack of ready access to the expertise required for such evaluations, the current prescribing information for the available EHL-FVIII products does not include individualized PK-based dosing [94], while the recommended fixed dose is based on individualized PK-guided dosing in some clinical trials [94]. Unlike hemophilia A, PK-guided prophylaxis has limited value in most adult patients with hemophilia B on standard FIX products [96,97] and PK-guided dosing strategies for EHL-rFIX products is considered likely to be challenging due to the inter-individual variability and complexity of FIX PKs and the uncertainty regarding the optimal sampling time that best accounts for a prolonged half-life [98]. Accordingly, population-based PK estimation with reduced plasma sampling is considered a more practical and less expensive PK-based estimate of factor requirements than an individualized approach in both hemophilia A and hemophilia B [94,98].

In addition, there are several challenges with PK-guided prophylaxis when EHL factor concentrates are used [99]. In contrast to available conventional FVIII products, which present approximately the same PK characteristics enabling similar treatment outcome when used interchangeably, EHL products demonstrate unique PKs resulting in different dose and dosing interval requirements and consequently variable treatment outcome [14]. Long-term outcome data are also lacking for using low ABR targets as a surrogate for preserved joint health in prophylaxis [19,100].

Given the unpredictable impact of bioengineered products on individual patients, increased knowledge on the PK parameters of new anti-hemophilic molecules with prolonged half-lives will improve tailored prophylaxis based on individual needs and PK characteristics, offering new possibilities for effective prophylaxis [17,19]. Population-based PK models using EHL factors will be available in the near future for routine clinical use to help guide PK tailoring [19].

## Challenges in Monitoring Treatment Via Laboratory Assays

Monitoring factor levels through laboratory assays is an important part of ensuring patient safety during hemophilia management. Commonly used laboratory assays for measuring FVIII or FIX activity may not be the optimal method for some EHL-rFVIII or -rFIX products, such as those modified through PEGylation or fusion to albumin or immunoglobulin [68,101].

While measurement of recombinant coagulation factor concentrates has always been complicated by the discordance between the measurements carried out with different types of assays [68], the molecular modifications applied to extend the half-lives of clotting factors lead to additional novel interactions with the reagents [68] and create new challenges for laboratories, especially those using one-stage assays to assess therapeutic efficacy [101]. Although it differs between different EHL products to what extent the accuracies of laboratory assays are affected (i.e. how well assay monitoring works) [68], the heterogeneity in assay monitoring is considered to be associated with clinically significant over- or underestimation of plasma factor concentration, which might have an adverse impact on the management of patients and might result in an unnecessary search for inhibitor antibodies [68,101].

Apart from the molecular modifications done to prolong the half-lives of the clotting factors, various properties of the original molecules themselves, as well as those of the assays, along with the use of different calibration methods contribute to the discrepancies in determining the plasma factor activity levels [68,102].

Chromogenic assays show less variability than one-stage assays in the measurement of FVIII activity levels, possibly due to a restricted choice of available assay kits and reagents [103,104]. Comparison among assays used for characterization of potency for various modified rFVIII products indicates that the results of chromogenic assays are more reliable across different kits [23,101,103,105].

Hence, chromogenic assays are considered as the assays of choice for monitoring patients treated with several EHL-rFVIII or -rFIX concentrates. However, there are several challenges associated with the implementation of chromogenic assays in routine clinical laboratory practice, including increased expenses and technical complexity as well as the higher inter-laboratory variability at very low factor levels when compared to one-stage assays [68,103,106].

Concordance between assays used in laboratories and by pharmaceutical companies to measure the potency of a product, effective communication between the laboratories and the clinicians, and conveyance of relevant information by companies for correct monitoring of their products to both local laboratories and clinicians are essential for proper monitoring [68,101]. Additional laboratory and clinical studies are required for optimization and standardization of the laboratory assays to correctly measure and monitor EHL concentrates [68].

## Conclusion

EHL factor concentrates have been shown to be well tolerated, safe, and efficacious in the treatment and prevention of bleeding episodes in people with hemophilia. These concentrates have the potential to induce higher trough levels with less frequent injections; thus, they reduce the infusion burden and facilitate adherence to prophylactic regimens [19,76,85]. Moreover, significant improvements in HRQoL have been shown with EHL factors in a large proportion of subjects, including those who have been on prophylaxis with SHL products. EHL products seem to have filled this gap by increasing adherence and further improving the HRQoL of hemophilia patients [87]. In addition to that, the usage of EHL factors in PTPs has not been associated with increased inhibitor formation. Results of clinical studies in PUPs and in the setting of ITI are eagerly awaited. However, available data with rFVIIIFc encourage the use of EHL products in ITI protocols to eradicate inhibitors, particularly in refractory patients and those with high-risk profiles [52,89].

Given the evolutions in the treatment of hemophilia in recent years with the advent of multiple non-replacement treatment options entering the market, clinicians and patients now face the prospect of having a variety of choices for individualizing treatments according to their needs [41]. EHL-rFIX and -rFVIII products have already become important alternatives in improving hemophilia care in clinical practice, while issues like the potential impact of different mechanisms of half-life prolongation on long-term safety (e.g., fusion technology versus PEGylation), cost-effectiveness, and immunogenicity in PUPs are yet to be clarified [29]. EHL factor concentrates will very soon be challenged by alternative products, including subcutaneous non-factor treatments like emicizumab or fitusiran and gene therapy. Exciting developments are about to occur in the near future of hemophilia treatment and we have to wait until all the battle lines are drawn and new options fall into place before discussing which one is optimal.

## Figures and Tables

**Table 1:**
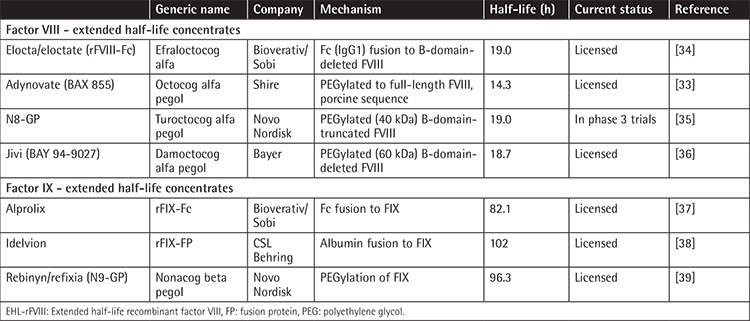
Currently available EHL-rFVIII and -rFIX concentrates [41].

**Table 2:**
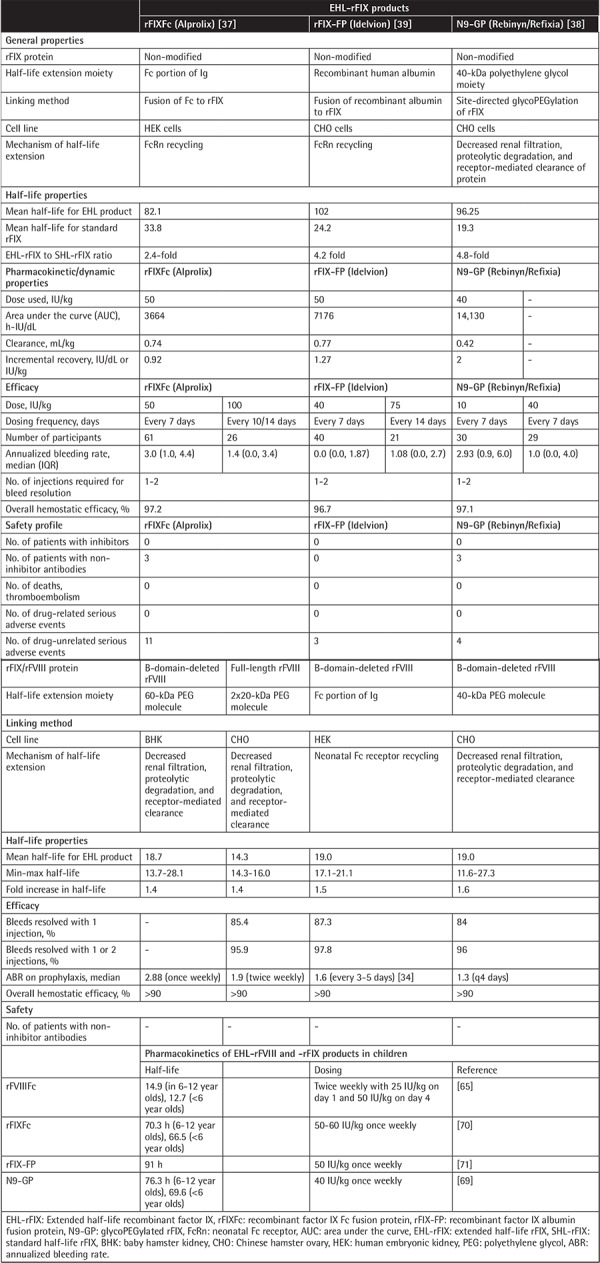
Characteristics of EHL-rFIX and -rFVIII products.
